# Epilepsy and Related Neuropsychiatric Comorbidities: Basic and Clinical Research

**DOI:** 10.2174/1570159X2108230510162504

**Published:** 2023-06-15

**Authors:** Rita Citraro, Antonio Leo

**Affiliations:** 1 Science of Health Department, “Magna Graecia” University of Catanzaro, Catanzaro, Italy

Epilepsy is one of the most frequently encountered neurological conditions worldwide. The International League Against Epilepsy (ILAE) pointed out that epilepsy is a disorder of the brain characterized not only by recurrent seizures but also by its neurobiological, cognitive, psychological, and social consequences, emphasizing the long-term psychosocial issues and the high prevalence of psychiatric and behavioral disorders that are frequently more harmful than seizures themselves, worsening the quality of life in patients with epilepsy (PWE) [1]. Several studies suggest that epilepsy and neuropsychiatric comorbidities have a particular bidirectional link, sharing common underlying mechanisms. To date, the relationship between epilepsy and neuropsychiatric disorders has been established not only for depression and anxiety but also for schizophrenia, dementia, and autism. Moreover, neuropsychiatric side effects can sometimes be secondary to antiseizure medications (ASMs). Direct medical costs in PWE are largely (80%) related to comorbidities rather than epilepsy [2, 3]. ASMs could suppress seizures in up to two-thirds of all patients, although the long-term prognosis is unaffected. Epilepsy surgery seems to be an effective non-pharmacological tool to reach long-term seizure freedom in selected patients with drug-resistant epilepsy, but it is probably underutilized. With increased knowledge of the gradual onset of epilepsy, epigenetic factors and pharmacogenomics become the hope for better, disease-modifying, or even curative, pharmacological, and non-pharmacological medication strategies [4].

This Research topic aimed to highlight basic, clinical, and translational research involved in studying epilepsy and its related neuropsychiatric disorders. A careful evaluation of all these aspects will contribute to optimizing therapy with a positive impact on seizure management, neuropsychiatric well-being, and quality of life in PWE.

The review by Neri *et al*. [5] discussed state of the art about seizures and epilepsy in stroke, cerebral hemorrhage, and leukoaraiosis. With an increasingly aging population, the increasing incidence and prevalence of epilepsy associated with cerebrovascular diseases are predictable [6]. According to the authors, to date, there are few predictors for both poststroke epilepsy (PSE) and early seizures (ES). However, among these, the severity and extension of stroke, cortical involvement, and hemorrhagic transformation have been identified as strong predictors for ES and/or PSE onset [7]. Moreover, current clinical studies do not support the hypothesis that antiseizure medications (ASMs) may counteract epileptogenesis in patients with cerebrovascular diseases.

Concerning pharmacological treatment, Operto *et al*. [8] provided a comprehensive overview of the main therapeutic tools available to manage epilepsy associated with intellectual disability, which remains a therapeutic challenge. The authors emphasized that, in addition to currently available ASMs, the ketogenic diet, hormone therapy, and surgery play a crucial role, especially in patients with drug-resistant epilepsy.

As widely reported in the literature, a PWE is more prone to develop psychiatric comorbidities, particularly depression . Such comorbidities are frequently more harmful to patients than seizures themselves, worsening their quality of life [2, 9]. Such a high frequency suggests that epilepsy and psychiatric disorders might share common pathological mechanisms. In agreement with the review by Pisani *et al*. [10], several aspects contribute to making the matter very complex from a therapeutic point of view. As affirmed in the review by Tallarico *et al*. [11], antidepressant drugs influence both depression and epilepsy. In fact, older generations of antidepressants, such as tricyclic, can reduce the seizure threshold, whereas preclinical and clinical evidence support that the newer antidepressants have less effect on neuronal excitability; they also reduce seizure severity. However, further clinical trials with appropriate protocols are needed to clarify the real contribution of these drugs in the management of PWE (besides their effects on mood) [12]. Not surprisingly, as discussed in the review by Zaccara and Franco [13], ASMs and drugs for psychiatric diseases are frequently used in combination. Accordingly, pharmacodynamic and pharmacokinetic interactions between these drugs must be considered, especially if they can lead to a change in seizure control and/or adverse drug reactions [14]. Clinically relevant pharmacokinetic interactions can be predicted by knowledge of CYP enzymes involved in the biotransformation of individual drugs [15]. In the clinical context, pharmacokinetic interactions can be assessed with drug monitoring and are consequently much better documented than pharmacodynamic interactions [10].

The review by Fortunato *et al*. [16] illustrated the emerging role of immunity and inflammation in a wide range of neurological disorders, including epilepsy and its associated psychiatric comorbidities. As reported, the knowledge of the mechanisms underpinning the complex interplay among autoimmunity, neuroinflammation, ictogenesis, and epileptogenesis could aid in the development of disease-modifying therapies [17]. The development of innovative ASMs, targeting the key pathological mechanisms also involved in neuroinflammation, was described in the review by Sciaccaluga *et al*.

The review by Rollo *et al*. [18] described the role of ASMs in migraine prophylaxis. Migraine is also a common comorbidity in PWE, and, not surprisingly, several ASMs, such as topiramate and valproic acid/valproate, have been shown to be effective in migraine prophylaxis. The authors pointed out the need to understand the antimigraine mechanisms of ASM, including their capacity to influence the pro-migraine calcitonin gene-related peptide CGRP signaling pathway to provide new innovative drugs for migraine/epilepsy treatment.

We, the Guest-Editors, would like to express our gratitude to the many authors who contributed to this special issue: Epilepsy and Related Neuropsychiatric Comorbidities: Basic and Clinical Research.
